# A broadly conserved fungal chorismate mutase targets the plant shikimate pathway to regulate salicylic acid production and other secondary metabolites

**DOI:** 10.1128/mbio.02031-25

**Published:** 2025-10-20

**Authors:** Dandan Shao, Nathaniel M. Westrick, Wende Liu, Oded Yarden, Jun Zhao, Yuri Kimura, Hiroshi Maeda, Damon L. Smith, Mehdi Kabbage, Martin B. Dickman

**Affiliations:** 1 Department of Plant Pathology, University of Wisconsin-Madison 5228, Madison, Wisconsin, USA; 2 Valley Laboratory, Connecticut Agricultural Experiment Station, Windsor, Connecticut, USA; 3 State Key Laboratory for Biology of Plant Diseases and Insect Pests, Institute of Plant Protection, Chinese Academy of Agricultural Sciences 243827 , Beijing, China; 4 Department of Plant Pathology and Microbiology, The R.H. Smith Faculty Agriculture, Food and Environment, The Hebrew University of Jerusalem 26742, Jerusalem, Israel; 5 Department of Horticultural and Plant Protection, Inner Mongolia Agriculture University 117454 , Hohhot, Inner Mongolia, China; 6 Department of Botany, University of Wisconsin-Madison 5228, Madison, Wisconsin, USA; 7 Institute for Plant Genomics and Biotechnology, Department of Plant Pathology and Microbiology, Texas A&M University 199053, College Station, Texas, USA; Cornell University, Ithaca, New York, USA

**Keywords:** plant-fungal interactions, plant shikimate pathway, necrotrophic effector, secreted chorismate mutase, salicylic acid regulation

## Abstract

The molecular dynamics of plant-pathogen interactions are complex, involving a constant defense race between host plants and pathogens. Plants deploy antimicrobial metabolites and physiological responses to inhibit pathogens, while pathogens counteract these defenses using secreted proteinaceous effectors. Chorismate mutases (CMs) are effectors largely studied in biotrophic fungi and nematodes for their ability to hijack the host shikimate pathway by diverting chorismate from salicylic acid (SA) biosynthesis, subsequently undermining SA-mediated defenses against biotrophic infection. While CMs have been primarily linked to biotrophy, we identified a novel bifunctional CM from the broad host range and predominantly necrotrophic pathogen *Sclerotinia sclerotiorum* and demonstrate that orthologs of this secreted CM are present in far more diverse fungal lifestyles than initially theorized. Unlike currently characterized secreted CMs, *S. sclerotiorum* CM (SsCM1) localizes to the plant chloroplast where it interacts with plant plastidic CMs. While SsCM1 is a functional, albeit weak CM, it contains a novel domain architecture of bacterial origin, including a putative isochorismate pyruvate lyase (IPL) domain. Interestingly, transient expression of SsCM1 *in planta* increases rather than decreases host SA levels. Our results indicate that secreted CMs are broadly conserved in many plant-associating fungi beyond canonical biotrophs and likely facilitate infection through a novel manipulation of the plant shikimate pathway that redirects the flow of this pathway toward increased SA production and away from the biosynthesis of downstream antimicrobial compounds. We propose that SsCM1-like effectors represent a novel class of secreted CMs that may be commonly utilized by plant-associated necrotrophs to achieve pathogenic success.

**IMPORTANCE:**

Microbial effectors are small secreted proteins that help pathogens establish disease within the host environment. In biotrophic fungi, secreted chorismate mutases (CMs) like Cmu1 suppress the production of salicylic acid (SA), a key plant hormone involved in resistance against biotrophic pathogens. Since Cmu1 and its homologs are exclusively found in biotrophic pathogens, secreted CMs have been considered a hallmark of biotrophy. Surprisingly, we identified a secreted CM, encoded by *SsCM1*, in the predominantly necrotrophic fungus *Sclerotinia sclerotiorum*. Structural and functional studies suggest *SsCM1* is likely a functional homolog acquired from bacteria and specifically acts to suppress the production of antimicrobial compounds that would otherwise enhance plant resistance to necrotrophs. Unlike Cmu1, *SsCM1* localizes to plastids, inversely regulates SA, and is conserved more broadly across the fungal kingdom. Thus, our findings reveal a new branch of plastid-localized CMs in necrotrophs, offering new avenues for the development of potential broad-spectrum antimicrobial treatments targeting this pathogen group.

## INTRODUCTION

*Sclerotinia sclerotiorum* is a cosmopolitan necrotrophic fungal pathogen that can infect over 600 plant species (1–3), causing annual multimillion-dollar yield losses on economically important crops (4). Virulence factors for this aggressive pathogen have largely focused on the phytotoxic compound oxalic acid (5–8) and cell wall-degrading enzymes (CWDEs) (9). However, recent bioinformatic and functional studies have identified other components that are involved in the pathogenic success of this pathogen, including small RNAs (10), detoxifying enzymes (11–14), and effectors (12, 15–25). Defined as small secreted, cysteine-rich, molecules that facilitate plant colonization, phytopathogen effectors have been widely studied for their roles in pathogenicity (26). During the past two decades, advances in genome sequencing and computational tools have enabled the discovery of effectors from a wide range of necrotrophic fungal pathogens. However, with a few exceptions, our understanding of these molecules in necrotrophs is limited, and much of what we know about effector biology has been chiefly described in (hemi)biotrophic pathogens (27).

The shikimate pathway is used by bacteria, archaea, fungi, and plants for the biosynthesis of carbocyclic aromatic compounds (28, 29). Chorismate, the common branch point for the production of these metabolites, is converted by chorismate mutases (CMs) into prephenate, an intermediate in the biosynthesis of the aromatic amino acids phenylalanine (Phe) and tyrosine (Tyr), and further downstream to a large number of secondary metabolites including phenylpropanoids and phytoalexins (29) (Fig. S1). Chorismate can also be converted to isochorismate, which serves as a precursor for the plant hormone salicylic acid (SA) (30) (Fig. S1). Some fungal pathogens have both endogenous and secreted CMs, the latter of which are required for full virulence in plants (31). Homologs in plant pathogenic nematodes have also been shown to be important pathogenicity determinants (32–35). These secreted CMs are proposed to contribute to virulence through the manipulation of the plant shikimate pathway and associated defense compounds. The key role of the phenolic hormone SA, a metabolite derived from the shikimate pathway, in the regulation of disease signaling upon pathogen attack is well established. In general, SA-dependent defense responses are often deployed against biotrophic pathogens, while jasmonic acid (JA-) and ethylene (ET-) dependent defenses commonly discourage pathogens with predominately necrotrophic lifestyles (36, 37). Unsurprisingly, many pathogens make use of effectors to interfere with phytohormone pathways to their own benefits (38). One such example is found in the smut fungus *Ustilago maydis,* which secretes a CM encoded by *cmu1* and is required for full virulence in maize (31). During infection, Cmu1 interacts with a plant cytoplasmic CM and facilitates the conversion of chorismate into prephenate, funneling chorismate away from the SA biosynthetic pathway, therefore restricting SA-mediated defenses against this biotrophic pathogen (31). Initial genome data showed that secreted CMs were largely associated with the biotrophic fungal lifestyle (31). However, more recent sequencing data suggest that CM-like effectors are more widely distributed in fungi with diverse lifestyles, including predominately necrotrophic organisms such as *S. sclerotiorum*.

In this manuscript, we describe a novel group of secreted CMs that possess a chloroplast-targeting peptide and have a unique CM domain with greater homology with the bifunctional isochorismate pyruvate lyase (IPL)/CM proteins found in prokaryotes. Using the *S. sclerotiorum* CM ortholog, SsCM1, we show that this protein functions through a mechanism that is distinct from that of the *U. maydis* Cmu1. We propose that after secretion from the pathogen, SsCM1 translocates to the chloroplast where it upregulates SA levels, therefore decreasing antagonistic JA-mediated defenses against *S. sclerotiorum* and presumably inducing SA-mediated cell death to the benefit of the pathogen. As predicted to have a structure more similar to that of prokaryotic isochorismate pyruvate lyase (IPL), SsCM1 likely increases SA levels in the host by acting directly as an IPL to synthesize SA. Additionally, SsCM1, by acting as a weak CM, possibly redirects the flow of the chorismate pathway to limit downstream antimicrobial compounds and induce SA levels. Collectively, these results suggest that these secreted SsCM1-like effectors use a distinct mechanism to facilitate successful plant infection.

## RESULTS

### SsCM1-like effectors are novel chorismate mutases likely arising from cross-kingdom horizontal gene transfer

**Fig 1:**
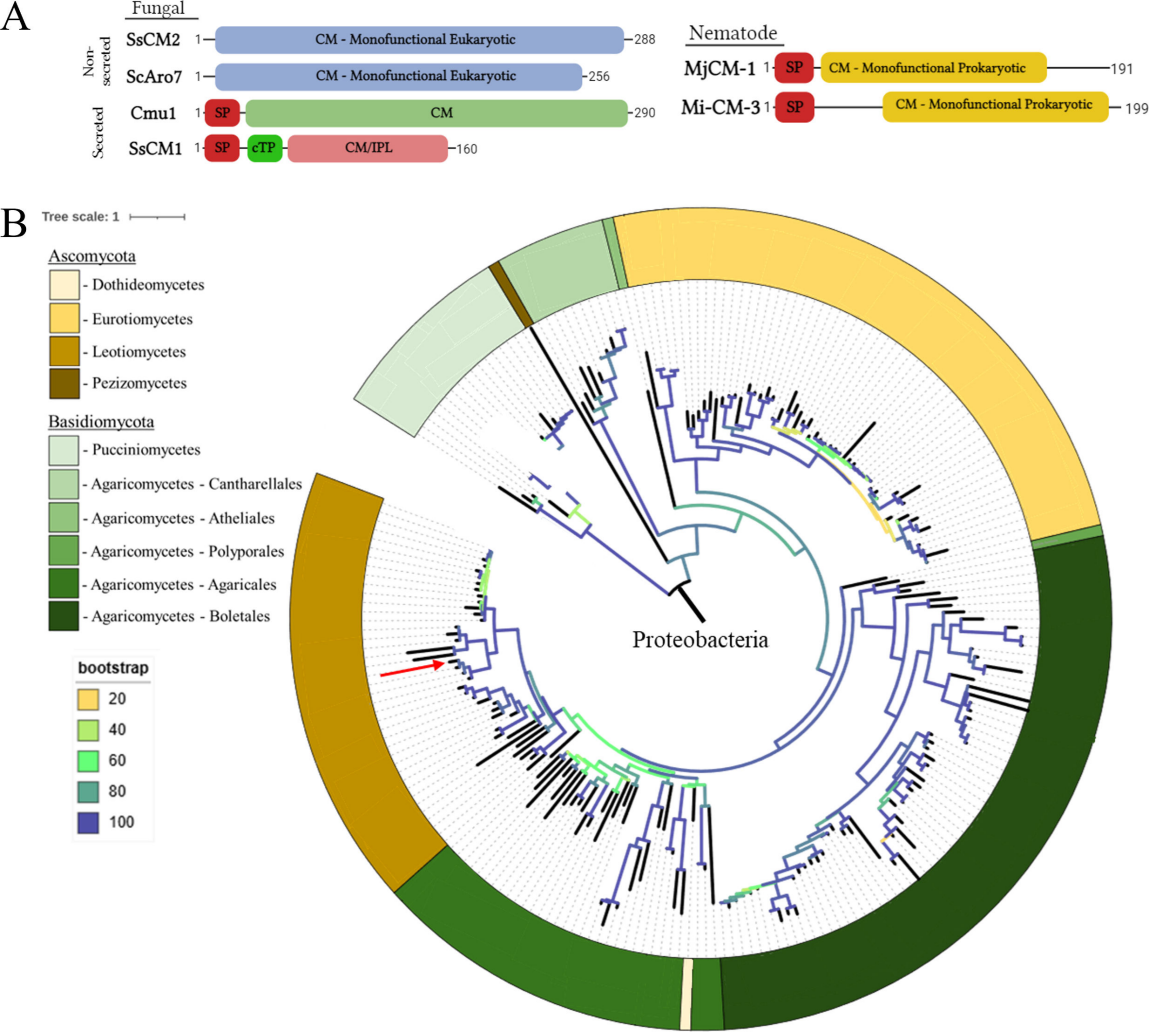
Domain architecture and phylogeny of select chorismate mutases (CMs). (**A**) Domain architecture of four fungal and two nematode-derived chorismate mutases, including the putative *Sclerotinia sclerotiorum-*secreted CM (SsCM1), *Ustilago maydis-*secreted CM (Cmu1), *Meloidogyne javanica-*secreted CM (MjCM-1) and *M. incognita* CM (Mi-CM-3), non-secreted SsCM2 from *S. sclerotiorum*, and non-secreted ScARO7 from *Saccharomyces cerevisiae*. SP, signal peptide; CM, monofunctional eukaryotic; CM, monofunctional prokaryotic; IPL, isochorismate pyruvate lyase; cTP, chloroplast targeting peptide. Numbers represent amino acid length. (**B**) Maximum likelihood phylogenetic tree of proteins showing homology to SsCM1. Tree was rooted in Proteobacteria, and bacterial sequences were collapsed. Branch colors represent bootstrapping confidence (20–100). Red arrow indicates SsCM1. Full species names are provided in Fig. S2 in the supplemental material.

Secreted CM effectors have been characterized in various plant pathogens including the fungus *U. maydis* (Cmu1) (31) and nematode *Meloidogyne incognita* (Mi-CM-3) (35), and it has been broadly assumed that these effectors are hallmarks of biotrophic plant-pathogen interactions given their canonical SA-suppressing activity (31, 35). To evaluate this, the predominately necrotrophic fungal pathogen *S. sclerotiorum* was analyzed for the presence of CM-like genes in its genome. Preliminary genomic analysis identified two putative CM genes within the *S. sclerotiorum* genome, hereupon referred to as *SsCM1* (XP_001584707.1) and *SsCM2* (XP_001590829.1). Protein homology and subcellular localization prediction suggest that SsCM2 is likely an endogenous, cytoplasmic CM, which has been shown in other species to play a pivotal role in the fungal shikimate pathway (39). Unlike SsCM2*,* however, SsCM1 contains a predicted signal peptide (SP) and an unusual CM domain, suggesting a functional divergence between the two genes (Fig. 1A). While SsCM2 contains a monofunctional CM domain (TIGR01802) found commonly in eukaryotes, SsCM1 has a CM domain containing a predicted isochorismate pyruvate lyase (IPL) motif (PRK07075), sharing greater homology with the bifunctional IPL/CM proteins found in prokaryotes (Fig. 1A) (40). Unlike CMs which convert chorismate into prephenate as a precursor to aromatic amino acid production, bacterial IPLs convert isochorismate directly into SA (41, 42). The evolutionary history of SsCM1 was inferred using the maximum likelihood method from an alignment of predicted SsCM1 homologs (Fig. 1B; Fig. S2). The vast majority of these homologs were proteobacteria, whereas proteins containing the IPL domain were comparatively uncommon in fungi. The tree was rooted within proteobacteria given the apparent ubiquity of IPL/CM proteins within the phylum, and the resulting tree suggests two distinct HGT events leading to the presence of *SsCM1* orthologs in a limited number of Eurotiomycete and Leotiomycete species; however, this pattern may alternatively reflect widespread gene loss following an earlier HGT event into a common ancestor within Ascomycota (Fig. 1B; Fig. S2).

As Cmu1, from the biotrophic pathogen *U. maydis*, is the only other well-characterized CM effector in fungi and is known to localize to the host cytoplasm, all predicted fungal SsCM1 homologs registered in NCBI were analyzed bioinformatically for the presence of both secretion SPs and any other predicted subcellular localization signals within plants (Table 1). Most of these homologs contained a predicted SP, but surprisingly, ~70% of Leotiomycete homologs, including *S. sclerotiorum*, also contained a predicted chloroplast-targeting peptide (cTP), a feature which was comparatively rare in other fungal groups (Fig. 1A; Table 1). Such localization has not been observed in the secreted CMs found in the biotrophic plant pathogens that have been evaluated to date (31, 32, 35). As the Leotiomycete species containing secreted CMs appear to share a common necrotrophic lifestyle, this subcellular localization may be relevant to this particular lifestyle. Taken together, SsCM1 appears to represent a novel class of secreted CM effectors present throughout the fungal kingdom and is found in fungi with lifestyles seemingly incompatible with the SA-suppressing activity of currently characterized CM effectors.

**TABLE 1:** Frequency of signal peptide (SP) /chloroplast targeting peptide (cTP) in SsCM1 homologs within Ascomycota

	Eurotiomycete	Leotiomycete	Dothideomycete	Pezizomycete
Total species	56	40	1	1
Frequency of predicted SP	89.29%	92.50%	100.00%	0.00%
Frequency of predicted cTP	3.57%	67.50%	100.00%	0.00%

### SsCM1 is secreted and translocates to the host chloroplast during infection

**Fig 2:**
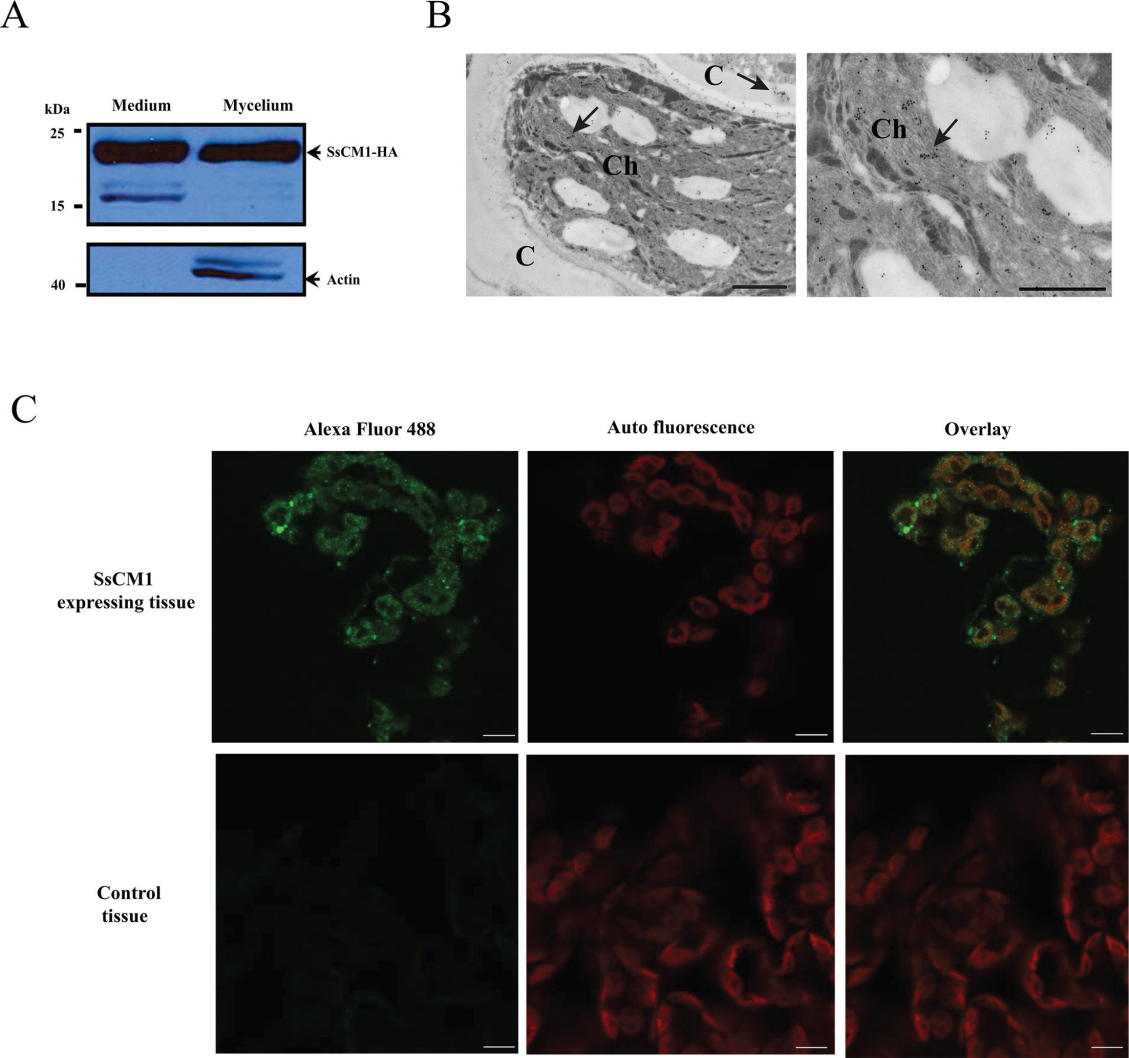
SsCM1 is secreted and localized to chloroplasts during infection. Crude proteins were extracted from the cultural medium (for secreted proteins) and fungal mycelium (for nonsecreted proteins) and were analyzed using Western blotting (**A**). HA-tagged SsCM1 was detected not only in the medium but also in fungal hyphae, while nonsecreted actin control was only present in fungal hyphae, indicating SsCM1 can be secreted from the fungus. (**B**) Subcellular localization of HA-tagged SsCM1 during infection of tomato plant was visualized by Immune-gold assay. As shown in the left and the right (zoomed-in view of left) panels where the plant tissue was infected by *Sclerotinia sclerotiorum* transformed with SsCM1-HA, SsCM1 signals, indicated by black arrows, are present in the chloroplast (Ch) and cytoplasm (**C**) during infection. (Scale bar: 0.5 µm.) (**C**) Fluorescence microscopy shows SsCM1 with cTP element transiently expressed in *Nicotiana benthamiana* leaves. Colocalization of fluorescence on SsCM1^ΔSP1-20^ conjugated with Alexa Fluor 488 is evident with the host chloroplast (red autofluorescence) (scale bar: 25 µm.)

As mentioned previously, SsCM1 is predicted to contain a secretion signal at the N-terminus with a strong prediction score (0.864) based on SignalP. To examine whether SsCM1 is indeed secreted, we used two approaches. We first generated a hemagglutinin (HA)-tagged SsCM1 strain followed by immunoblotting. SsCM1-HA was clearly detected in minimal media supernatant under standard *in vitro* growth conditions (Fig. 2A), with fungal actin serving as the control. The *in-planta* subcellular localization of SsCM1 during infection was also analyzed given the predicted cTP (Fig. 1). *S. sclerotiorum* expressing HA-tagged *SsCM1* was inoculated onto 4-week-old *N. benthamiana* leaves. Immunogold labeling of SsCM1-HA showed that signals were observed in the host cytoplasm and chloroplasts (Fig. 2B). The functional relevance of the cTP was further examined by immunofluorescent microscopy. SsCM1-HA expressed in *N. benthamiana* was detected by Alexa Fluor 488-conjugated HA antibody and visualized using confocal microscopy (Fig. 2C). Green fluorescence from Alexa Fluor 488 associated with SsCM1 was largely colocalized with chloroplast autofluorescence, in accordance with the transmission electron microscopy (TEM[1][2]ata) (Fig. 2B). Therefore, the cTP of SsCM1 is clearly relevant to the subcellular localization of this protein within the host environment. Together, these results indicate that SsCM1 is a secreted protein that translocates into the plant chloroplasts upon infection.

### SsCM1 enhances salicylic acid levels in plants

**Fig 3:**
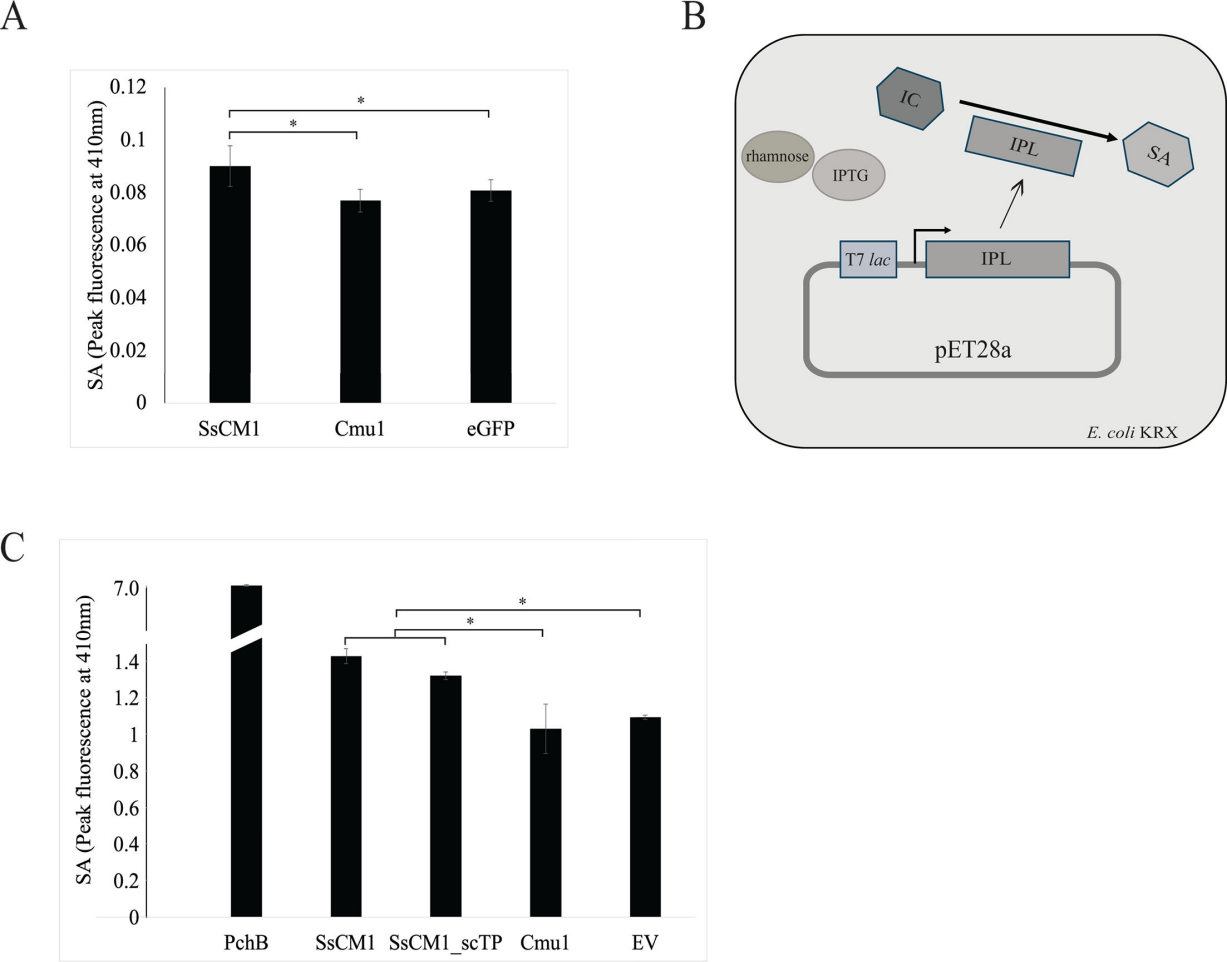
Overexpression of SsCM1 upregulates SA in *Nicotiana benthamiana,* possibly through IPL activity. (**A**) Relative abundance of SA in *N. benthamiana,* which transiently expresses SsCM1, Cmu1, or GFP through *Agrobacterium*-mediated transformation. Constructs pGWB402Ω containing SsCM1, Cmu1, or eGFP were agro-infiltrated into *N. benthamiana*. Three days after inoculation, SA was extracted from leaves and analyzed through HPLC. (**B**) Schematic representation of functional IPL assay in *Escherichia coli* KRX containing the T7 promoter-driven pET28a plasmid. Upon induction by rhamnose and IPTG, the gene encoding a functional IPL is expressed and converts endogenous isochorismate (IC) to SA. (Graph was modified from reference 44.) (**C**) Relative quantity of SA in *Escherichia coli* expressing SsCM1, SsCM1-scTP, Cmu1, PchB, and EV. *E. coli* with pET28a carrying SsCM1^ΔSP/cTP1-66^, SsCM1-scTP^ΔSP/shorter cTP1-53^, Cmu1^ΔSP1-21^, PchB, or EV were cultured in LB to reach an OD_600_ of 0.5. Bacterial cultures were added with IPTG and rhamnose with final concentrations of 0.2 mM and 0.1% wt/vol, respectively, and shaken at 275 rpm, 24°C for overnight to induce the expression of proteins. SA was extracted from 1 mL of the bacterial culture and analyzed by HPLC. (*: *P* value < 0.05 level.) All treatments represent a minimum of three replicates.

We further explored how the chloroplast localization underlies the mode of action of SsCM1. As indicated by bioinformatic analyses (Fig. 1A), *S. sclerotiorum SsCM1* potentially encodes a protein with both CM and IPL activities. IPLs are known to convert isochorismate into salicylate in bacteria (41, 42). In plants, SA is an important defense signal and mediates resistance against phytopathogens (36). Two biosynthetic pathways for SA are proposed; one is isochorismate synthase (ICS) pathway and another is phenylalanine ammonia-lyase (PAL) pathway. The first step of the former occurs in the plastids (43) (Fig. S1). In alignment with the chloroplast localization (Fig. 2), it is conceivable that SsCM1 acts as an IPL to directly convert isochorismate to SA, which is then released into the cytoplasm to mediate downstream processes (43). Alternatively, SsCM1 can also act as a CM, as previously described in the *U. maydis* Cmu1 (30) and potentially affect the conversion of chorismate into prephenate and downstream phenylpropanoids. To verify if the SA level in plants is impacted by the presence of SsCM1, we expressed SsCM1 in *N. benthamiana* using an *Agrobacterium*-mediated transient expression system. Three days after Agro-infiltration, leaves were collected and analyzed for their SA content using HPLC. Compared to Cmu1 and eGFP control, SsCM1 expression resulted in a significantly higher SA content (Fig. 3A). Interestingly, this is in contrast to Cmu1, which was shown to prevent SA accumulation upon pathogen attack (31). Thus, we propose that SsCM1 acts in a mechanism that is distinct from that used by Cmu1 and likely functions to increase SA levels in the host upon pathogen challenge.

### SsCM1-mediated accumulation of salicylic acid is driven by its IPL/CM bifunctionality

Whether the induction of SA is driven by IPL or CM activities of SsCM1 was further investigated. To test the IPL activity of the protein, *SsCM1* was cloned upstream of a rhamnose-inducible promoter and transformed into *Escherichia coli* KRX strain (Fig. 3B). *E. coli* is known to utilize isochorismate synthase encoded by *entC* to synthesize isochorismate but lacks the ability to convert isochorismate into SA (45). Upon induction by rhamnose and isopropyl-β-d-thiogalactopyranoside (IPTG), a protein with IPL activity is expressed in *E. coli* and is expected to convert endogenous isochorismate into SA, which can be further detected using HPLC. PchB, a known *Pseudomonas aeruginosa* IPL (41), was used as the positive control, while Cmu1 lacking the IPL domain was used as a negative control in this experiment. As expected, a strong SA signal was detected from *E. coli* cells expressing PchB, whereas those of Cmu1 only had a background signal equivalent to the empty vector control (Fig. 3C). Intriguingly, SA levels in SsCM1 expressing *E. coli* were statistically higher than those of Cmu1- expressing cells or those containing the empty vector control (Fig. 3C). These data demonstrate that SsCM1 possesses some IPL activity when expressed in *E. coli*.

**Fig 4:**
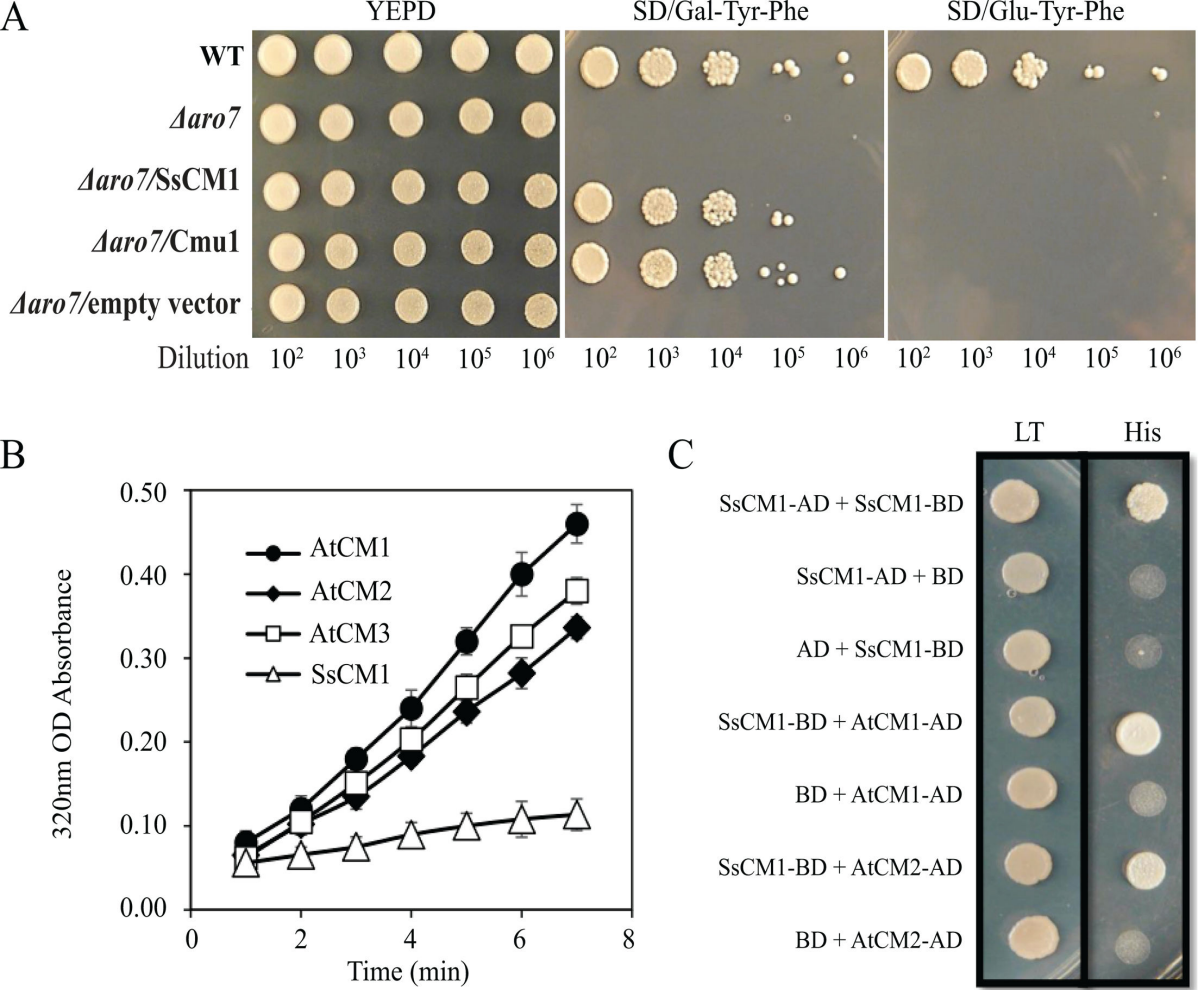
SsCM1 interacts with plant host chorismate mutases (CMs), therefore attenuating CM activity toward the phenylpropanoid pathway. Biochemical function of SsCM1 catalytic activity was explored by using bio-function complementary assay (**A**). The yeast *Δaro7* mutant, *Δaro7* complemented by SsCM1 (*Δaro7/*SsCM1), Cmu1(*Δaro7/*Cmu1), or empty vector (*Δaro7/*empty vector), respectively, was cultured on the aromatic amino acid-null medium (-Tyr and -Phe) with supplementation of glucose (Glu) or galactose (Gal). Biochemical function of *in vitro* expressed SsCM1 and Arabidopsis CMs was carried out (**B**). Enzymatic activity of CMs was analyzed by their capability of converting chorismate into prephenate, which is then automatically converted into phenylpyruvic acid with high absorbance in 320 nm wavelength. Binding assay between SsCM1 and Arabidopsis CM (AtCM1 and AtCM2) was performed *in vitro* (**C**) by Yeast-2-Hybrid assay.

To explore whether SsCM1 has dual functions as predicted (Fig. 1A), we also evaluated the CM activity of SsCM1 by testing the ability of SsCM1 to complement the yeast CM mutant Δ*aro7. Saccharomyces cerevisiae* Aro7 shares similarities with SsCM1 in the CM catalytic domain (Fig. 1A) and is required for the biosynthesis of the aromatic amino acids Phe and Tyr (46). *S. cerevisiae* mutants lacking *Aro7* are unable to grow on media lacking these two aromatic amino acids (Fig. 4A). However, the overexpression of SsCM1 in the Δ*aro7* yeast strains restored the ability of these mutants to grow in media lacking Phe and Tyr (Fig. 4A). The CM activity of SsCM1 was also determined using purified recombinant His-tagged protein and by running *in vitro* enzymatic assays that monitored the conversion of chorismate to prephenate (31, 47). Accordingly, both the yeast complementation and enzymatic assays indicate that the *SsCM*1 gene encodes an active functional chorismate mutase (Fig. 4A and B). Interestingly, however, *in vitro* CM enzymatic activity assays showed that although all tested chorismate mutases are able to convert chorismate into prephenate, the catalytic capacity of SsCM1 is much weaker compared to that of *Arabidopsis* chorismate mutases (~16% of the activity of AtCM1) (Fig. 4B) and *U. maydis* Cmu1 (Fig S3). This suggests that SsCM1 is less efficient in converting chorismate than its plant and *U. maydis* counterparts.

Chorismate mutases are known to exist as dimers. Thus, we reasoned that SsCM1, due to its weak CM activity, may interfere with plant CMs through competitive binding. We next tested the ability of SsCM1 to bind *Arabidopsis* CMs in yeast. The *Arabidopsis* genome contains three predicted chorismate mutase genes—the chloroplast-localized *AtCM1* and *AtCM3*, and one cytosolic chorismate mutase *AtCM2* (48). We cloned *AtCM*1 (chloroplast representative) and *AtCM*2 (cytosol representative) individually into a prey yeast vector, while SsCM1 was used as bait. Results showed that SsCM1 binds not only to chloroplast-localized AtCM1 but also to the cytoplasmic AtCM2 (Fig. 4C), though the mechanistic relevance of AtCM2-SsCM1 interaction requires further investigation. Together, we propose that SsCM1 likely acts as a weak CM to slow down the flow of chorismate to prephenate, thus funneling more chorismate toward SA biosynthesis. As upregulation of SA often leads to the downregulation of JA responses (36) and likely increased cell death, the manipulation of SsCM1 of plant CM activity is expected to contribute to the pathogenic development of *S. sclerotiorum* and potentially other pathogens with SsCM1-like effectors.

### Structural analysis revealed SsCM1 shared high similarity with PchB, but not Cmu1

**Fig 5:**
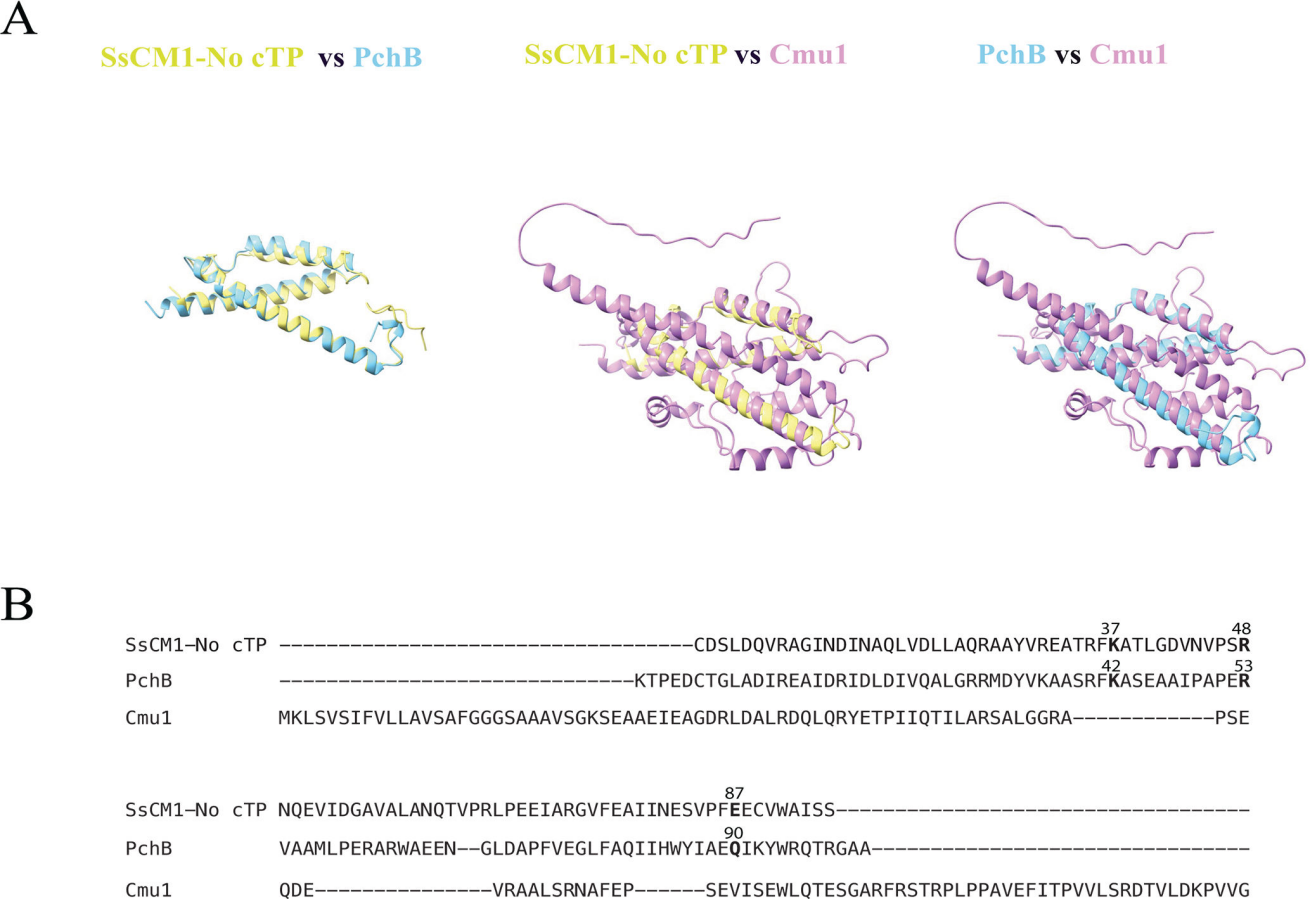
SsCM1 shares low sequence identity, yet high structural similarities, with PchB. Superposed structures (**A**) and sequence alignment (**B**) were generated by pairwise structural alignments between *Sclerotinia sclerotiorum-*secreted chorismate mutase without chloroplast-targeting peptide (SsCM1-No cTP), *Pseudomonas aeruginosa* isochorismate pyruvate lyase (PchB), and *Ustilago maydis-*secreted CM (Cmu1) by RCSB.org.

As the structural conformation of proteins is strongly related to their physiological and biological functions, the protein structures and relevant functions of SsCM1, PchB in *P. aeruginosa*, and Cmu1 in *U. maydis* were also analyzed. Structural Protein Data Bank (PDB) formatted structures of SsCM1, PchB, and Cmu1 were first predicted by AlphaFold2 using MMseqs2 (49) and then compared with each other using Pairwise structure alignments (50, 51). Chorismate mutases from *A. thaliana* (AtCM1) and *E. coli* (EcCM) were also included in the analyses. As shown in Table 2, the structural alignment between SsCM1 and PchB is stronger than that between SsCM1 and Cmu1, as indicated by a lower root mean square deviation (RMSD) (1.56 vs 3.13). Consistently, template modeling (TM) score (52) of the SsCM1-PchB pair is greater than 0.5 (0.81), whereas the SsCM1-Cmu1 pair has a TM score below 0.5 (0.27), suggesting that SsCM1 adopts the same protein folding as PchB but not Cmu1. Despite only 26% amino acid sequence identity between SsCM1 and PchB, their structural similarity suggests these two proteins likely possess analogous functions as IPL enzymes. Results showed that SsCM1 shares low structural similarity with chorismate mutases from *A. thaliana* and *E. coli*, as evidenced by their low TM scores and high RMSD values seen in these paired comparisons (Table 2), suggesting SsCM1 is distinct from plant or bacterial CMs. Further, superposed structures revealed that SsCM1 exhibits similar folding and α-helix arrangements to PchB (Fig. 5A). Prior studies identified that amino acids lysine (K) 42, arginine (R) 53, and glutamic acid (Q) 90 are critical to PchB function (53). While residues K42 (K37) and R53 (R48) are conserved in SsCM1, Q90 (E87) is substituted with glutamate (E) (Fig. 5B). As suggested by Künzler et al. (53), glutamic acid (Q) at position 90 in PchB is critical for IPL activity, and the substitution of this residue with glutamate (E) reduces the enzymatic activity by ~80%. This Q to E substitution might partially explain the modest IPL activity of SsCM1 in *E. coli* compared to PchB (Fig. 3). Taken together, these predictions support that SsCM1 is a structural and functional equivalent of PchB, though with weaker IPL activity, at least in a bacterial environment.

**TABLE 2:** Pairwise structure alignment for *Sclerotinia sclerotiorum* secreted chorismate mutase without chloroplast targeting peptide (SsCM1-No cTP), *Pseudomonas aeruginosa* (PchB), *Ustilago maydis* secreted CM (Cmu1), *Arabidopsis thaliana* chorismate mutase (AtCM1), and *Escherichia coli* chorismate mutase/prephenate dehydratase (EcCM)

Entry	RMSD^*a*^	TM-score^*b*^	Identity^*c*^	Equivalent residues^*d*^	Sequence length^*e*^	Modeled residues^*f*^
SsCM1	–^*g*^	–	–	–	95	95
SsCM1 vs PchB	1.56	0.81	26%	93	101	101
SsCM1 vs Cmu1	3.13	0.27	8%	93	290	290
SsCM1 vs AtCM1	2.92	0.23	11%	93	340	340
SsCM1 vs EcCM	2.32	0.22	21%	92	386	386

^*a*^ RMSD (root mean square deviation): the value is calculated by the distance (Å) of the backbone carbon-alpha atoms between aligned pairs in superposed structures. A lower value indicates a better alignment between structures.

^*b*^ TM-score (template modeling score): the value measures the topological structural similarities between the template and models (52) and ranges between 0 and 1. Unrelated proteins have scores < 0.2. Proteins with same folding have scores >0.5.

^*c*^ Identity (sequence identity percentage): the value is calculated by the percentage of the identical sequence in paired residues.

^*d*^ Equivalent residues: the number structurally equivalent residues in the paired alignment.

^*e*^ Sequence length: the number of residues in the deposited sequence.

^*f*^ Modeled residues: the number of residues used for structural alignment.

^*g*^ –, not applicable.

### *SsCM1* is plant-induced, but the inactivation of SsCM1 does not impact fungal development and disease outcomes.

**Fig 6:**
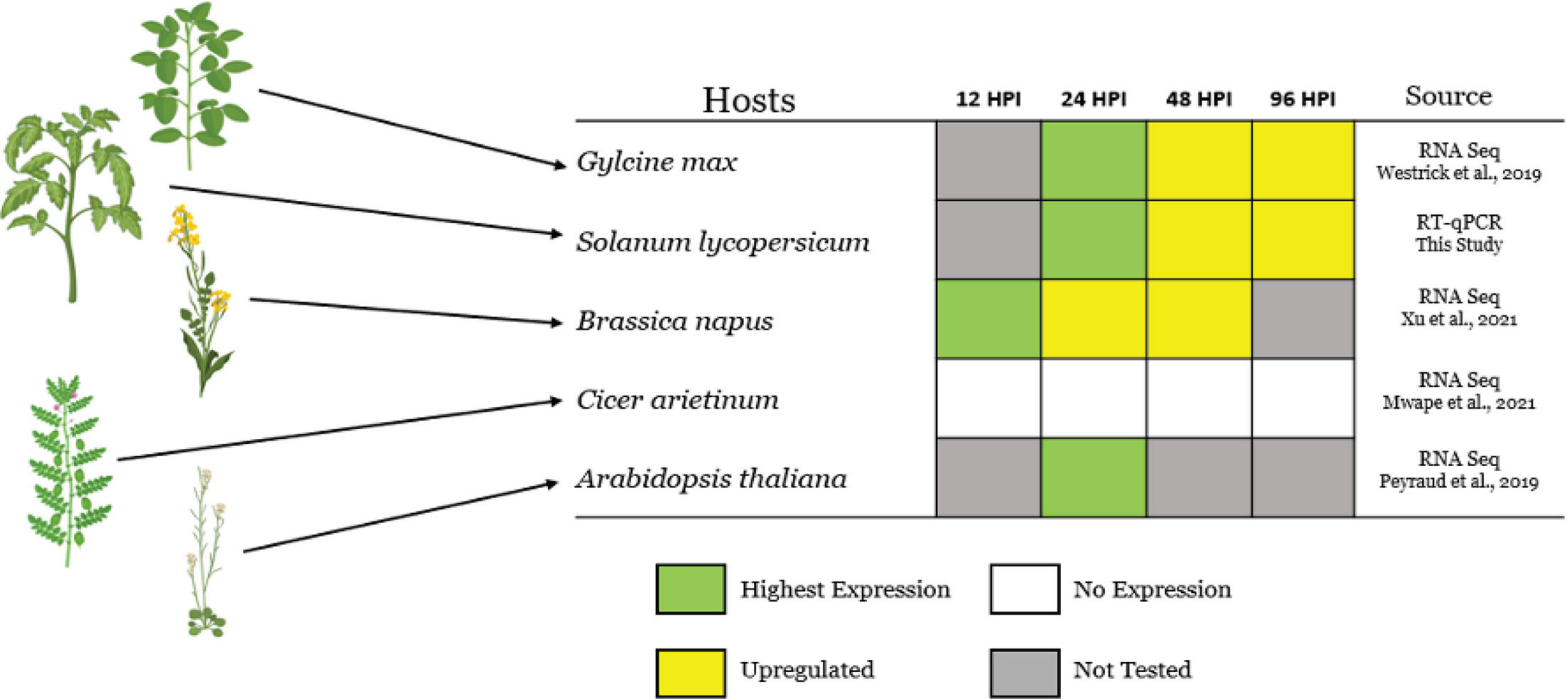
Expression of *SsCM1* in planta. This figure depicts a simplified collation of *SsCM1* expression patterns from across multiple hosts, sourced from either existing transcriptomic data sets or RT-qPCR conducted in this study. All gene expression changes are in reference to RNA extracted from *in vitro* controls, and colors denote expression patterns. Green: the timepoint with the highest expression level identified for SsCM1 in a given study, Yellow: a timepoint in which SsCM1 was upregulated in-planta to a lesser extent than that denoted in green boxes, White: a timepoint in which SsCM1 was not upregulated during infection, Gray: a timepoint in which gene expression was not evaluated for a given host.

As mentioned, effectors are low-molecular weight plant-induced secreted molecules that modulate host responses. The expression patterns of *SsCM1* during infection were evaluated in available databases in a number of plant species, including soybean (*Glycine max*) (12), tomato (*Solanum lycopersicum*) (Fig. S4), canola (*Brassica napus*) (19), *Arabidopsis thaliana* (11), and chickpea (*Cicer arietinum*) (54). In these studies, *SsCM1* expression was upregulated at different stages of infection in most species, with *C. arietinum* as an exception (Fig. 6), suggesting that *S. sclerotiorum* upregulates *SsCM1* expression in a host-specific manner.

To investigate the role of SsCM1 during infection, the wild-type *SsCM1* gene was replaced by a hygromycin phosphotransferase (Hyg) cassette via CRISPR/Cas9 combined with homologous recombination. Two independent knockout mutants (∆*SsCM*1-1 and ∆*SsCM*1-2) were generated. No developmental defects were observed in the generated mutants *in vitro* (data not shown). When wild-type (strain 1980) and corresponding ∆*SsCM*1 mutants were inoculated on detached leaves of soybean and *N. benthamiana*, similar disease levels were observed with both strains (Fig. S5). Analysis of lesion development found no statistical difference between mutants and wild-type (Fig. S5). While this lack of phenotype is surprising, *S. sclerotiorum* is known to possess a large repertoire of secreted effectors and detox components targeting the shikimate pathway (12–14). This suggests that a functional redundancy between SsCM1 and other virulence determinants may explain this lack of phenotype.

## DISCUSSION

The shikimate pathway is utilized by bacteria, archaea, fungi, and plants for the production of aromatic amino acids and other compounds that are essential for development, immune responses, and other processes (28, 29). Chorismate is the intermediate branch point of the pathway, and its partitioning toward various products is mediated by several chorismate metabolizing enzymes including chorismate mutases (CM), which convert chorismate to prephenate. While endogenous, nonsecreted CMs are broadly conserved in organisms that utilize the shikimate pathway, such as bacteria (55, 56) and fungi (31), secreted CMs are found in certain phytopathogenic fungi and nematodes (31–35). Moreover, many of the above-secreted CMs discovered in phytopathogenic organisms are shown to be involved in pathogenicity/virulence (31–35). The importance of secreted CM effectors in pathogenicity is highlighted by their independent acquisition by phytopathogens through a number of distinct evolutionary paths. Both SsCM1-like effectors and the secreted CMs found in phytopathogenic nematodes are the likely result of HGT from bacteria, whereas the secreted CM from the biotrophic pathogen *U. maydis*, Cmu1, is presumably the result of a gene duplication from an endogenous CM (Fig. 1A and B) (31, 35). While the presence of secreted CM effectors has traditionally been considered a hallmark of biotrophic interactions, our phylogenetic analysis places SsCM1 homologs within the genomes of a number of biotrophic, necrotrophic, and even saprotrophic fungi, suggesting a more complex interaction with the host than previously thought (Fig. 1B; Fig. S2). The chloroplastic localization of SsCM1 and other secreted CMs from related Leotiomycetes is a relatively unique feature of these proteins when compared to other characterized CM effectors (31, 32, 35) and homologs throughout the fungal kingdom (Table 1). The apparent bifunctionality of SsCM1 along with these differences in localization may help explain its presence in the genomes of fungi with such a wide range of lifestyles, including the biotrophic *Puccinia* spp., necrotrophic *Rhizoctonia* spp., and saprotrophic Agaricomycetes (Fig. S2). Differences in IPL vs CM activity, mixed with the cytoplasmic vs chloroplastic localization, would likely yield a wide range of outcomes with differential value across the range of plant-fungal interactions. Whether these differences in localization specifically contribute to the diverging functionality of SsCM1 compared to other CMs, such as those found in smut fungi, will still need to be explored.

During the investigation of the specific roles of SsCM1, the *in-planta* induction of SsCM1 in *S. sclerotiorum* suggested the protein might be beneficial for the infection process or required by the pathogen in response to a nutrient-limiting host environment (Fig. S4). Disease assays on a few plant hosts did not reveal an obvious contribution for SsCM1 during infection (Fig. S5), raising the possibility that SsCM1 may be important for other hosts not examined in this study, may be required under specific environmental conditions, or may serve functions beyond pathogenesis. Future experiments, such as infection assays on plants expressing SsCM1, could provide a better understanding of its role in pathogenicity. Nevertheless, SsCM1 could still facilitate infection as part of a broad, multi-pronged strategy by *S. sclerotiorum* to target the shikimate pathway. Indeed, our previous work showed that *S. sclerotiorum* uses multiple tools to target the shikimate pathway, including efficient detox mechanisms (13, 14). Despite sequence variability and the presence of a cTP, based on their predicted function, it is reasonable to assume that most CM-encoding effectors contribute to virulence by targeting the chorismate branch of the shikimate pathway, in particular, by modulating the production of SA. SA is well known for its pivotal role in plant immunity. In general, SA mediates the plant’s defense response against pathogens with biotrophic lifestyles, in contrast to JA, which is involved in defense against necrotrophic pathogens (36). Decades of research have been dedicated to investigating how plants biosynthesize SA. More recently, researchers revealed that in higher plants, pathogen-induced SA is derived from chorismate through biosynthesis routes that occur in both plastids and cytoplasm (43). Specifically, upon pathogen attack, plastidic chorismate is converted into isochorismate by isochorismate synthases and further released into the cytosol through the chloroplast envelope localized transporter (43, 57). Instead of deploying IPL-like enzymes akin to SA-producing bacteria (41, 42), plants like *Arabidopsis* convert cytosolic isochorismate into SA through a two-step biosynthesis pathway, which involves isochorismoyl-glutamate synthase and isochorismoyl-glutamate A pyruvoyl-glutamate lyase (58). Components of the SA biosynthesis pathway can potentially be targeted by pathogen virulence factors. For example, isochorismate was shown to be targeted by effectors from oomycete pathogen *Phytophthora sojae* and the fungus *Verticillium dahliae*, which are encoded by *Pslsc1* and *Vdlsc1*, respectively. Specifically, these two effectors function as isochorismatase to hydrolyze isochorismate into 2,3-dihydro-2,3-dihydrobenzoate, therefore reducing isochorismate-deriving SA and its downstream defense responses (59). As the CM domain in SsCM1 is predicted to encode an isochorismate pyruvate lyase (IPL) (Fig. 1A), SsCM1 is also likely to catalyze isochorismate as the substrate. Although no *bona fide* IPLs were identified in plants (44), expressing bacterial IPLs has been shown to successfully upregulate SA expression in plants (58, 60). Similar to phenotypes seen from plants expressing bacterial IPLs, overexpression of SsCM1 in *N. benthamiana* also resulted in SA upregulation (Fig. 3A). To verify if SsCM1 is indeed an IPL, we further expressed SsCM1 in *E. coli*, which has isochorismate but lacks IPL, and detected significantly higher levels of SA production above background (Fig. 3C). This enzymatic activity was further supported by structural analyses of SsCM1, which showed that SsCM1 shared significant structural similarities, despite low sequence identity, with *P. aeruginosa* PchB having IPL activity, but not Cmu1 from *U. maydis* (Fig. 3A; Table 2).

SsCM1 is not an unusual example among fungal effectors that share structural and functional commonalities with other proteins despite low sequence homologies (61). To date, five classes of sequence-unrelated structurally similar (SUSS) effectors have been identified, including MAX-effectors (Magnaporthe Avrs and ToxB-like) in *Magnaporthe oryzae* (62, 63), RNase-like Proteins associated with Haustoria (RALPH) effectors in *Blumeria graminis* (64, 65), LARS (Leptosphaeria Avirulence and Suppressing) effectors in *Leptosphaeria maculans* (66), ToxA-like effectors (67) and FOLD (Fol dual-domain) effectors (68) in *Fusarium oxysporum*. It is suggested that these SUSS effectors may have evolved from common ancestors with conserved structural folds (61, 69). Similarly, *S. sclerotiorum* may have acquired SsCM1 vertically from fungal ancestors that originally gained IPL horizontally from bacteria. Under selection pressure, this IPL-like protein likely underwent rapid sequence divergence to evade immune recognition or adapt to a particular biological system, while maintaining its original function. We propose that SsCM1 may function as an IPL during *S. sclerotiorum*-plant interactions to increase SA levels in the host and create an environment that is conducive to disease establishment. Although *S. sclerotiorum* is thought to exhibit a short biotrophic phase during the early stages of infection (70), this effector is more likely involved in supporting its primarily necrotrophic mode of infection.

The CM activity of the bifunctional domain of SsCM1 is also of interest and suggests that this effector has chorismate mutase activity. Indeed, yeast complementation assays showed that SsCM1 is able to complement the *S. cerevisiae aro7* mutant and restore yeast growth on media lacking Phe and Tyr (Fig. 4A). Interestingly, similar to its modest IPL activity, the CM activity of SsCM1 is much weaker than that of plant CMs (Fig. 4B) or even *U. maydis* Cmu1 (Fig. S3). This is not surprising as competitive binding to two distinct substrates is not uncommon in bifunctional proteins with proximal or shared active sites, reducing the efficiency of both enzymatic activities (71). However, this weak CM activity may have major consequences in a host environment. Using confocal and electron microscopy, we show that SsCM1 translocates to chloroplasts during infection (Fig. 2), whereas Cmu1 acts in plant cytoplasm (31). The deregulation of CM activity and cytosolic location of Cmu1 are crucial for mode of action as Cmu1 dimerizes with plant non-allosteric cytoplasmic CM (ZmCM2), diverting chorismate away from plastidic SA biosynthesis (31). In contrast, our study shows *S. sclerotiorum* SsCM1 translocates to chloroplasts (Fig. 2), where it interacts with plant CM (AtCM1) as shown in Fig. 4C. Since chorismate is mainly produced in plastids (29, 72), the metabolic disruption caused by SsCM1 is likely to be more pronounced at the location where its target substrates predominantly accumulate. Heterodimers composed of enzymatically inactive and active CM monomers have been shown to exert dominant-negative effects (31), suggesting the enzymatically inefficient SsCM1 might form a heterodimer with AtCM1, reducing the activity of the native plant CM. Alternatively, SsCM1 could potentially disrupt plant CMs by competitively binding to host chorismate, thereby slowing the conversion of chorismate to prephenate. Manipulating plant CM through either mechanism could increase chorismate availability for SA biosynthesis, leading to the upregulation of SA levels, as seen in Fig. 3A. Additionally, SsCM1 interference in the conversion of chorismate to prephenate could also influence the production of secondary metabolites derived from this pathway, including antimicrobial compounds like phenylpropanoids, which play a documented role in plant defense (73, 74). In fact, our previous work suggests a strong correlation between the upregulation of phenylpropanoid intermediates and resistance to *S. sclerotiorum* (73).

 Taken together, since SsCM1 homologs are widely conserved across the fungal kingdom, we propose that SsCM1-like effectors constitute a novel class of CM effectors. These effectors hijack the plant’s shikimate pathway to elevate SA levels within the host while simultaneously reducing the biosynthesis of defense-related antimicrobial compounds, ultimately facilitating disease establishment.

## MATERIALS AND METHODS

### Plant and fungal materials

*S. sclerotiorum* wild-type (1980) and transformants were maintained at room temperature on potato dextrose agar (PDA). *Glycine max* (William 82), *N. benthamiana,* and tomato (*Solanum lycopersicum*) plants were grown and maintained under standard growth chamber conditions (5).

### Pathogenicity assays

Pathogenicity assays were conducted as previously described (5). Leaves of assigned plants were excised and inoculated with 5 mm PDA plugs containing actively growing *S. sclerotiorum* wild-type isolate (1980) and Δ*SsCM1* mutants. At 1, 2, 3, or 4 days post-inoculation (dpi), infected leaves were photographed to evaluate symptom development.

### Bioinformatic analyses

Signal peptide prediction was performed using the program of SignalP 4.0 (https://services.healthtech.dtu.dk/services/SignalP-3.0/). Chloroplast targeting elements were identified using the Chlorop 1.1 Server (https://services.healthtech.dtu.dk/services/ChloroP-1.1/). The phylogenetic tree of fungal CM domains was generated through the maximum likelihood method in IQTree using the K2*P* + G4 substitution model and 100,000 bootstrapping iterations. All CM homologs identified through a BLASTp search of the GenBank RefSeq database were included in the analysis. Tertiary structure for the chorismate mutase domain was modeled using the Phyre2 program (http://www.sbg.bio.ic.ac.uk/phyre2/). Structural Protein Data Bank (PDB) formatted structures of SsCM1, PchB, and Cmu1 were predicted by AlphaFold2 using MMseqs2 (49). Pairwise structure alignments for PDB structures were conducted by RCSB.org (50, 51).

### *Sscm1* gene replacement

The Δ*sscm1* mutants were generated using a modified form of the previously developed CRISPR/Cas9 split-marker-deletion approach (13, 14). Briefly, split markers targeting *Sscm1* were generated using PCR to amplify 500–600  bp regions upstream (Sscm1-LF-F and Sscm1-LF-R) and downstream (Sscm1-RF-F and Sscm1-RF-R) regions of Sscm1. These amplicons were designed to contain 20  bp homology in the 5′ and 3′ regions of the hygromycin resistance cassette (HygR; 1.8  kb) found in pCRISPR-Cas9-TrpC-Hyg (75). The HygR cassette was amplified using PCR before being combined with the two flanking regions through fusion PCR, as described previously (76). Split markers were generated from this product by using overlapping primers within HygR (Hyg Split F and Hyg Split R) in conjunction with Sscm1-RF-F and Sscm1-LF-R, yielding two amplicons with an overlapping region of ~400  bp.

Two small guide RNAs (sgRNAs) targeting *Sscm1* were designed using the E-CRISP Design Tool (http://www.e-crisp.org/) and then subsequently generated using the GenCrispr sgRNA Screening Kit (L00689; Genscript Biotech Corp.). Both sgRNA and Alt-R S.p. Cas9 nuclease 3NLS (1081058; IDT) were diluted to a concentration of 4  µM and combined at a 1.2-to-1 ratio (3.6 µL of sgRNA to 3 µL of Cas9 protein) and incubated at room temperature for 5  min to assemble the RNP complex. These complexes were combined with 1  µg of each split-marker and transfected into *S. sclerotiorum* protoplasts using the polyethylene glycol (PEG) transformation described previously (77). Transformants were recovered and evaluated using the protocol described previously (78).

### SsCM1 secretion and subcellular localization assays

*S. sclerotiorum* strain SsCM1-HA was generated by transformation with plasmid pWL73 (SsCM1-HA driven by *Aspergillus nidulans* trpC promoter) into the wild-type fungal strain 1980. To analyze SsCM1-HA secretion, protein extracts of mycelia and liquid culture supernatants after precipitation with trichloroacetic acid were used for Western blot analysis with mouse-anti HA (Roche) and mouse anti-α-actin antibodies (Sigma).

To determine the location of SsCM1, *N. benthamiana* leaves were inoculated with fresh *S. sclerotinia* SsCM1-HA strain. The immunogold labeling assay for SsCM1-HA was performed as described previously (79).

The subcellular localization of SsCM1 was also confirmed by immunofluorescent microscopy. SP truncated SsCM1 was inserted into the pEarleyGate 301 vector containing HA tag using the Gateway Recombination Cloning kit (Invitrogen) and then transformed into *A. tumefaciens* GV3101. Resultant *A. tumefaciens* strains were infiltrated into 4- to 5-week-old *N. benthamiana* leaves using methods as described previously (80). Immunofluorescence using HA antibody conjugated with Alexa Fluor 488 (Thermo Fisher Scientific) was conducted according to the manufacturer’s instructions. Fluorescence was then examined and photographed under the Olympus IX81 inverted fluorescence microscopy with the GFP filter sets (excitation at 460–490 nm, emission at 510 nm), as described previously (5, 79).

### Quantitative real-time PCR

RNA was extracted from mycelia grown in PDB culture as well as from infected tomato plants at the indicated time points with the RNeasy Plant Mini Kit (Qiagen), treated with DNase (Qiagen), and subsequently used for cDNA synthesis. Quantitative real-time PCR was conducted as described previously (79). All reactions were performed with a minimum of three biological replicates. Relative *SsCM1* expression levels were calculated in relation to the values obtained for the constitutively expressed actin gene of *S. sclerotiorum* (Fig. S4).

### Yeast assays

SsCM1 interacting proteins were assayed *in vitro* by using the yeast-two hybrid system from Clontech Co. (Clontech, CA). *Arabidopsis* chorismate mutase genes (*AtCM1* and *AtCM2*) were amplified from an *Arabidopsis* cDNA library and introduced into the yeast prey vector pGADT7. Fungal chorismate mutase gene *SsCM*1 was cloned into both the prey (pGADT7) and bait (pGBKT7) vectors. The prey and bait constructs were confirmed by sequencing and co-transformed into yeast strain HF7C by Alkali-Cation yeast transformation (MP Biomedicals, OH). Trp^+^ and Leu^+^ transformants were identified and assayed for growth on SD-Trp-Leu-His medium. LacZ reporter gene expression and analysis were provided by Clontech Co. (CA). Empty vector plasmids encoding only the GAL4-activation or -binding domain (AD or BD), respectively, served as negative controls.

Yeast wild-type strain Y054679 and the *aro7* mutant (kindly provided by Dr. Regine Kahmann) were used in a complementation assay, as described previously (31). The *aro7* mutant strain was transformed with the corresponding pYES260 derivative constructs using the alkali-cation yeast transformation kit as mentioned above and tested for growth on medium lacking Phe and Tyr as described previously (31).

### Enzyme assays for chorismate mutase activity

*In vitro* enzymatic activity assay for CM activity was used to determine the ability of a given CM to convert chorismate into prephenate as described previously (31, 47). In detail, SsCM1, Cmu1, and *Arabidopsis* AtCM1 to AtCM3 were cloned into the pET expression system (Sigma-Aldrich) and expressed in *E. coli* BL 21 (47). His-tagged proteins were enriched using nickel-based affinity chromatography with Ni-NTA resin according to the manufacturer (Thermo Fisher Scientific). Enzymatic activities of CMs were assessed following procedures in (31). The conversion of prephenate to phenylpyruvate was measured as absorbance at 320 nm, and the rate of reaction was determined by plotting absorbance increase over time (minutes). Data represent the mean ± standard deviation of three technical replicates. As a negative control, protein purified from *E. coli* containing empty His-tag expression vector was included, and background values were subtracted from those of tested samples.

### Quantification of salicylic acid in *N. benthamiana*

To determine the effect of SsCM1 on plant SA level, SsCM1 lacking SP, Cmu1 without SP, and control eGFP were inserted into *A. tumefaciens* vector pGWB402Ω driven by CaMV35S promoter. Recombinant vectors were then transformed into *A. tumefaciens* GV3101 and infiltrated into 4- to 5-week-old *N. benthamiana*. As suggested by Goodspeed et al. (81), SA accumulates at the highest level at night in *Arabidopsis*; therefore, leaf samples were collected at night for a better representation of the SA quantity. Three days after infiltration, approximately 200 mg of infiltrated leaf tissue per sample and six replicates per treatment were collected in liquid nitrogen during the middle of the night cycle. Leaves were homogenized into fine powder by homogenizer at 1,500 rpm for 15 sec, and SA from the leaf powder was extracted by homogenizing with 600 µL of 65% methanol (vol/vol) at 1,500 rpm for 10 mins (MiniG, SPEX Sample Prep). SA from samples was then dried by speedvac and resuspended in 150 µL 65% methanol (vol/vol). One hundred μL of the suspension per sample was analyzed by HPLC (1200 Infinitely Series - Infinitely better, Agilent Technologies). Analytical conditions were as follows: column, Atlantis T3 C18 column (3 µm, 2.1 × 150 mm, Waters); solvent system, solvent A (water including 0.1% [vol/vol] formic acid) and solvent B (acetonitrile including 0.1% [vol/vol] formic acid); gradient program: 95% A/5% B at 0 min, 95% A/5% B at 5 min, 90% A/10% B at 8 min, 70% A/30%B at 10 min, 30% A/70% B at 15 min, 10% A/90% B at 18 min, 10% A/90% B at 20 min, 95% A/5%B at 20.5 min, 95% A/5%B at 25 min; flow rate: 0.3 mL/min; FLD: 300 nm of excitation, 410 nm of emission.

### Quantification of salicylic acid in *E. coli*

To examine the IPL activity, tested proteins were cloned into T7 promoter-driven pET28a plasmid in *E. coli* KRX cells according to the manufacturer’s protocol (Promega). Mature SsCM1 without cTP (Δ1-66 AA, based on the ChloroP prediction) or SsCM1 without shorter cTP (Δ1-53 AA) (predicted by alignment with other prokaryotic homologs from NCBI multiple alignment excluding eukaryotes, data not shown), SP-null Cmu1 (Δ1-21 AA), or PchB from *P. aeruginosa* were synthesized with codon-optimized (IDT) and expressed in *E. coli* KRX. *E. coli* with pET28a carrying SsCM1^ΔSP/cTP1-66^, SsCM1-scTP ^ΔSP/shorter cTP1-53^, Cmu1 ^ΔSP1-21^, PchB, or EV were cultured in LB to reach OD_600_ of 0.5. Bacterial cultures were added with IPTG and rhamnose with final concentrations of 0.2 mM and 0.1% wt/vol, respectively, and shaken at 275 rpm, 24°C for overnight to induce the expression of proteins. SA was then extracted from 1 mL of bacterial culture as described previously (42, 42), with slight modifications. In detail, 1 mL of bacterial culture was acidified to pH 2, and SA was extracted with chloroform (vol/vol of culture supernatant:chloroform, 1:0.5) upon vigorous shaking. The chloroform layer at the lower phase was then generated by centrifugation and collected into a new tube. Chloroform extraction was repeated three times, and SA in the lower phase was dried by speedvac. Pellets containing SA were then resuspended with 100 µL of 65% methanol (vol/vol), and SA quantity was analyzed by HPLC as described in methods for plant SA quantification.

## SUPPLEMENTAL MATERIAL

Supplemental material mbio.02031-25-s0001.docx Fig. S1 and S3 to S5, Table S1, and legends for Fig. S2.

Fig S2 mbio.02031-25-s0002.pdf Maximum likelihood phylogenetic tree of proteins homologous to SsCM1 with full species names.

ASM does not own the copyrights to Supplemental Material that may be linked to, or accessed through, an article. The authors have granted ASM a non-exclusive, world-wide license to publish the Supplemental Material files. Please contact the corresponding author directly for reuse.
