# Supplementary material for: A broadly conserved fungal chorismate mutase targets the plant shikimate pathway to regulate salicylic acid production and other secondary metabolites
Source: mBio. 2025 Oct 20;16(11):e02031-25. doi: 10.1128/mbio.02031-25 (PMC12607891; doi:10.1128/mbio.02031-25)
Supplement: Supplemental material — Fig. S1 and S3 to S5, Table S1, and legends for Fig. S2. [file mbio.02031-25-s0001.docx]

## **SUPPLEMENTARY FIGURES AND TABLES**

### **Figure S1. A schematic diagram of the shikimate pathway of plants.**

### ****

### **Figure S1: A schematic diagram of the shikimate pathway of plants.** Chorimsate mutase (CM) catalyzes the conversion of chorismate to prephenate, a key step in the shikimate pathway. ICS: isochorimate synthase; PAL: phenylalanine ammonia-lyase.

**Figure S2: Maximum likelihood phylogenetic tree of proteins homologous to SsCM1 with full species names.**

Due to the size limit, this figure is provided as a separate high-resolution file, Supplementary Figure 2.

**Figure S2:** **Maximum likelihood phylogenetic tree of proteins homologous to SsCM1 with full species names.** This tree corresponds to Figure 1B, but with all species names shown in full. The tree was rooted in Proteobacteria and bacterial sequences were collapsed. Branch colors represent bootstrap support values (20–100). The red arrow indicates SsCM1.

### **Figure S3.** SsCM1 displays weaker chorismate mutase activity in comparison to Cmu1.

**Figure S3:** **SsCM1 displays weaker chorismate mutase activity in comparison to Cmu1.** Enzymatic activity of *in vitro* expressed SsCM1 and Cmu1 were analyzed by their capacity to convert chorismate into prephenate, then to phenylpyruvic acid, which has high absorbance at 320nm wavelength.

### **Figure S4.** SsCM1 is plant induced during the early stages of infection.

**Figure S4. *SsCM1* is induced during infection of tomato.** (A) PDA plugs from actively growing cultures of *S. sclerotiorum* were inoculated onto fully expanded tomato leaves. The inoculated leaves were sampled and photographed 18, 24, 30, 48, 72 and 96 hours after inoculation. (B) Transcripts of SsCM1 were analyzed in mycelium and sclerotia harvested from rich media (PDB or PDA, respectively), as well as in fungus-infected tissue collected 18, 24, 30, 48, 72 and 96 hours after inoculation. The relative expression levels of SsCM1 were calculated in relation to the fungal actin gene. (C) Expression levels of *SsCM1* in mycelium from PDB were compared to those in minimal medium (MM) at 0, 8 and 16 hours after being transferred from original medium to PDB or MM, respectively. All the statistical data represent a minimum 3 replicates.

### **Figure S5.** Inactivation of *SsCM1* did not impact fungal virulence.


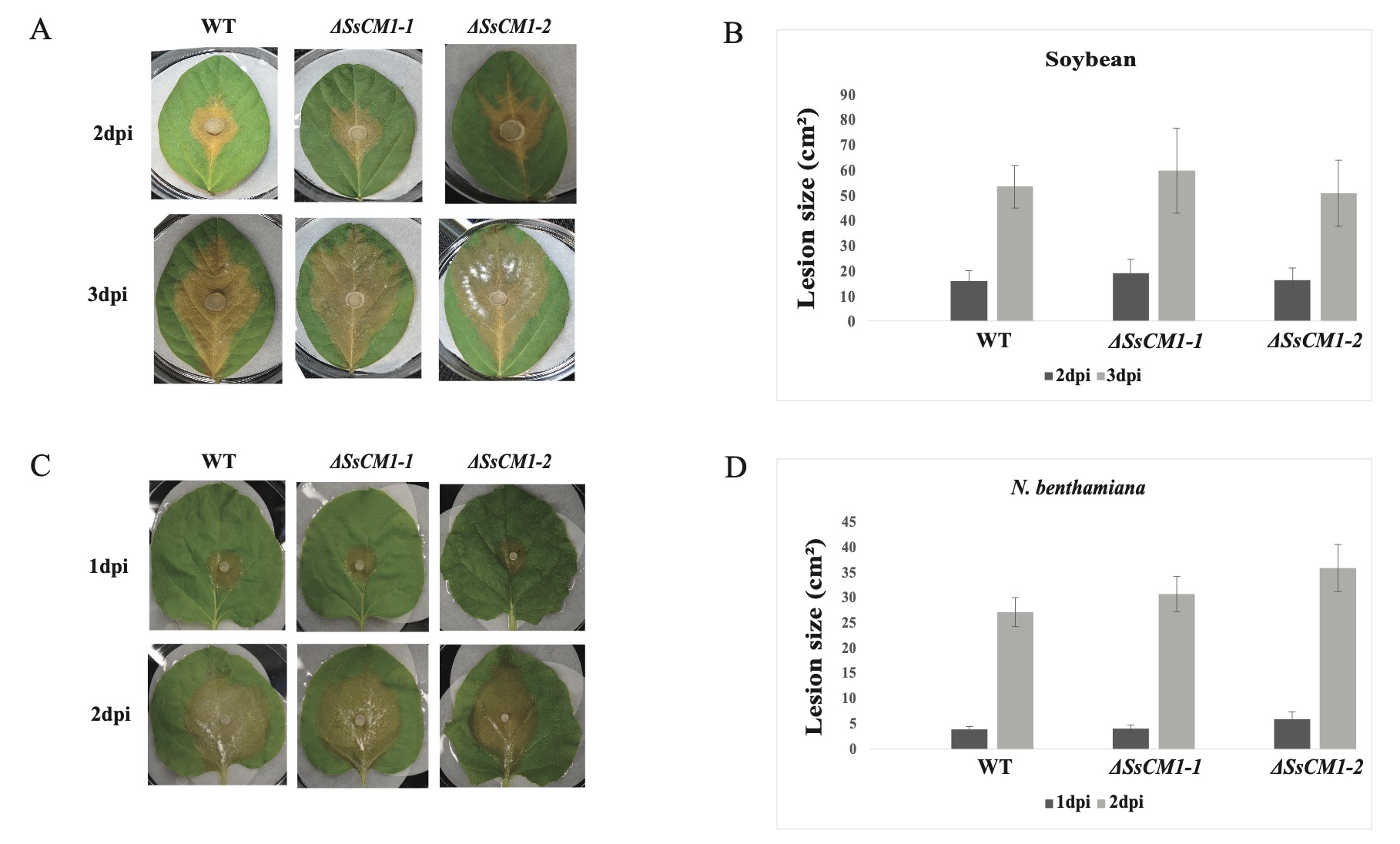


**Figure S5. Inactivation of *SsCM1* did not impact fungal virulence.** Lesions on the detached leaves of soybean (A, B) and tobacco (C, D) following inoculation of the following *S. sclerotiorum* strains (*SsCM1* null mutants: *ΔSsCM1-1 and ΔSsCM1-2; S. sclerotiorum* wild-type (WT)) were photographed 1, 2 and 3 days post inoculation (dpi) and analyzed with a minimum of 3 replicates (*P* value > 0.05).

###

### **Table S1.** Primers used in this study.

| Name | Sequence (5’-3’) | Use |
| --- | --- | --- |
| Sscm1-LF-F | CCATATTGAACATATGAAGATCGGT | Split markers targeting *Sscm1* |
| Sscm1-LF-R | TCTAAACAAGTGTACCTGTGTGATTTAGTCGATGTTCGCT | Split markers targeting *Sscm1* |
| Sscm1-RF-F | AATCCAATGCGTCTAGAGGGTGGGAATGAAGGGATGAAGA | Split markers targeting *Sscm1* |
| Sscm1-RF-R | CTTCTTTGGGCGAAGTGTATAC | Split markers targeting *Sscm1* |
| SsCM1-sgRNA1 | CCTCtaatacgactcactataGGTTGTGGCGGAGATGGCGAGgtttaagagctatgc | Small guide RNAs) targeting *Sscm1* |
| SsCM1-sgRNA2 | CCTCtaatacgactcactataGGCATGTTGTTCCATTAGGAAgtttaagagctatgc | Small guide RNAs) targeting *Sscm1* |
| attB_SsCM1_F | GGGGACAAGTTTGTACAAAAAAGCAGGCTatgaaattcaccaccatttccca | Expression in pGWB402Ω |
| attB2_SsCM1_R | GGGGACCACTTTGTACAAGAAAGCTGGGTCagaagaaatcgcccaaacaca | Expression in pGWB402Ω |
| attB_Cmu1_F | GGGGACAAGTTTGTACAAAAAAGCAGGCTatgaagttgagcgtgtccat | Expression in pGWB402Ω |
| attB_Cmu1_R | GGGGACCACTTTGTACAAGAAAGCTGGGTCggtgcacttgttggcgtg | Expression in pGWB402Ω |
| attB1_GFP_F | GGGGACAAGTTTGTACAAAAAAGCAGGCTATGGTGAGCAAGGGCGAG | Expression in pGWB402Ω |
| attB2_GFP_R | GGGGACCACTTTGTACAAGAAAGCTGGGTCCTTGTACAGCTCGTCCATGC | Expression in pGWB402Ω |
| SsCM1_IF_F | CGCGCGGCAGCCATATGTGCGATTCACTGGATCAAGTC | Expression in pET28a |
| SsCM1_IF_R | GCTCGAATTCGGATCCTTAGCTGCTTATCGCCCAAACAC | Expression in pET28a |
| SsCM1 scTP_IF_F | CGCGCGGCAGCCATATGTGCGATAGCCTGGATCAAGTCC | Expression in pET28a |
| SsCM1 scTP_IF_R | GCTCGAATTCGGATCCTTAGCTCGAAATCGCCCAGACAC | Expression in pET28a |
| Cmu1_IF_F | CGCGCGGCAGCCATATGGCGGCCGTAAGCGGCAAG | Expression in pET28a |
| Cmu1_IF_R | GCTCGAATTCGGATCCTTAGGTGCACTTATTGGCGTGGTC | Expression in pET28a |
| PchB_IF_F | CGCGCGGCAGCCATATGAAAACTCCCGAAGACTGCACCG | Expression in pET28a |
| PchB_IF_R | GCTCGAATTCGGATCCTCATGCGGCACCCCGTGTCTGG | Expression in pET28a |
