# Supplementary material for: A broadly conserved fungal chorismate mutase targets the plant shikimate pathway to regulate salicylic acid production and other secondary metabolites
Source: mBio. 2025 Oct 20;16(11):e02031-25. doi: 10.1128/mbio.02031-25 (PMC12607891; doi:10.1128/mbio.02031-25)
Supplement: Fig S2 — Maximum likelihood phylogenetic tree of proteins homologous to SsCM1 with full species names. [file mbio.02031-25-s0002.pdf]

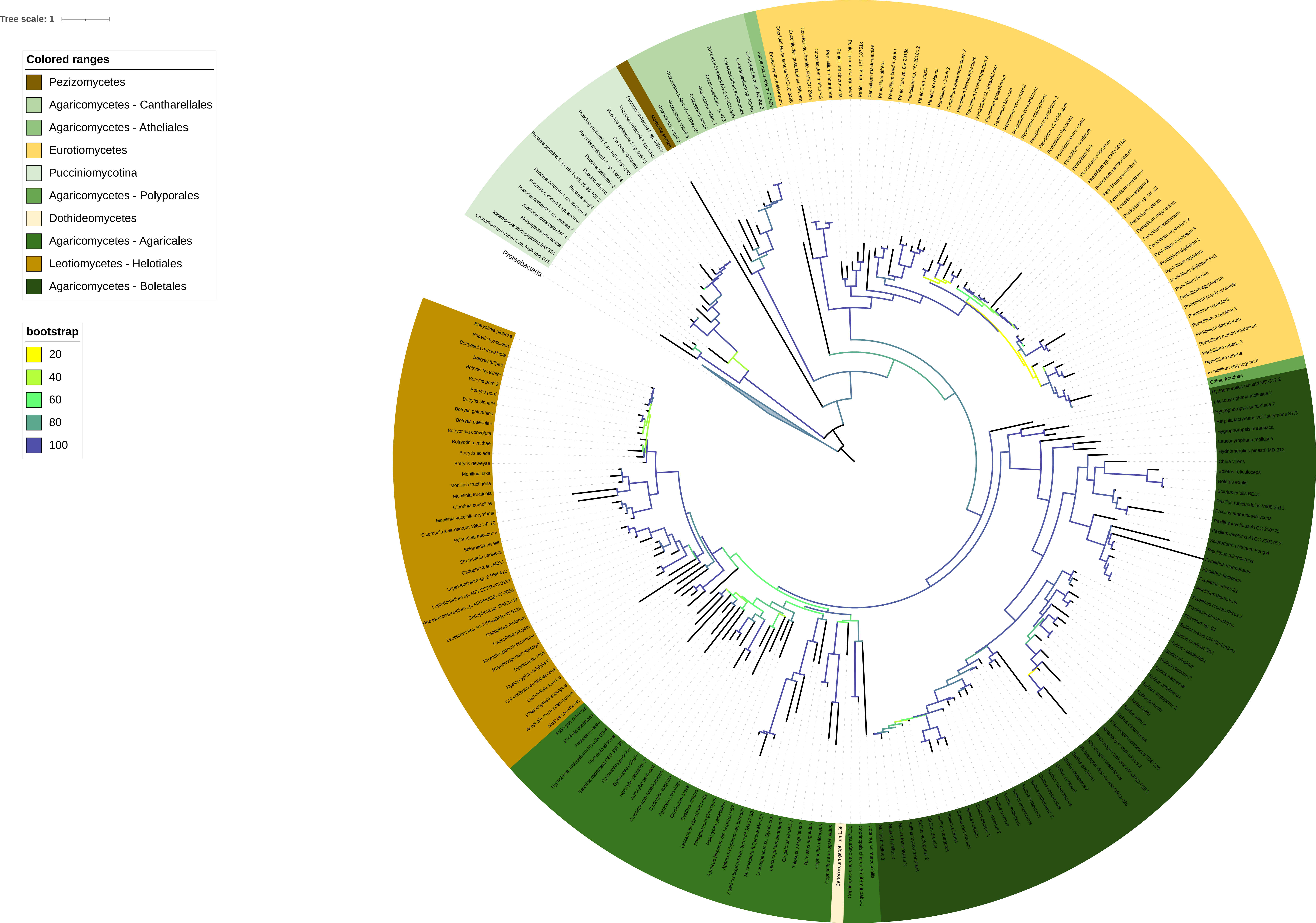

Tree scale: 1
Colored ranges
Pezizomycetes
Agaricomycetes - Cantharellales
Agaricomycetes - Atheliales
Eurotiomycetes
Pucciniomycotina
Agaricomycetes - Polyporales
Dothideomycetes
Agaricomycetes - Agaricales
Leotiomycetes - Helotiales
Agaricomycetes - Boletales
bootstrap
20
40
60
80
100
Proteobacteria
